# Vibration, and temperature run-to-failure dataset of ball bearing for prognostics

**DOI:** 10.1016/j.dib.2024.110403

**Published:** 2024-04-10

**Authors:** Wonho Jung, Sung-Hyun Yun, Yong-Hwa Park

**Affiliations:** Center for Noise and Vibration Control Plus, Department of Mechanical Engineering, Korea Advanced Institute of Science and Technology, 291, Daehak-ro, Yuseong-gu, Daejeon 34141, Republic of Korea

**Keywords:** Bearing, Fatigue life, Acceleration test, Condition monitoring, Fault prediction

## Abstract

Condition based maintenance (CBM) has become a very important issue in the industry because it can decrease the inventory as the need of parts can be planned by the identification of a potential failure. However, in order to predict the life span of the ball bearing, it is necessary to acquire data according to the all life span of the bearing. This article presents the time-series dataset, including vibration, and temperature, of the ball bearing under run-to-failure. Through the accelerated life test, the ball bearing was failed at 128 working hours, and the vibration and temperature data for the all running section were included. The type of fault was identified through microscopic analysis of the damaged ball bearing. The established dataset can be used to verify newly developed state-of-the-art methods for prognosis the remaining useful life (RUL) of the ball bearing. Mendeley Data. DOI: 10.17632/5hcdd3tdvb.6

Specifications Table

**Table:** 

Subject	Engineering – Mechanical Engineering
Specific subject area	Rotating Machine Condition Monitoring
Type of data	Table, Image, Figure Raw, Processed
Data collection	The dataset was acquired from the testbed that can make accelerated life test. For the accelerated life test, an axial load of 300 kg (2.94 kN) and a vertical load of 600 kg (5.88 kN) were applied to the ball bearing. By repeating 5 min of operation and 15 min of rest, the rotating speed was maintained at 1770 ∼ 1780 RPM until the bearing failed. The accelerated bearing life test was terminated when the bearing temperature exceeded 85 °C (Celsius) and the vibration value exceeded 9 m/s^2^.
Data source location	• Institution: Human Lab., Center for Noise and Vibration Control Plus, Department of Mechanical Engineering, Korea Advanced Institute of Science and Technology (KAIST) • City: Daejeon • Country: South Korea
Data accessibility	Repository name: Ball Bearing Vibration, and Temperature Run-to-Failure Dataset Data identification number: 10.17632/5hcdd3tdvb.6 Direct URL to data: https://data.mendeley.com/datasets/5hcdd3tdvb/6 Instructions for accessing these data: Access to website

## 1 Value of the Data

• To estimate the remaining useful life of the ball bearing, we need to assume the data trends. Therefore, many kinds of research are assumed to be Weibull distributed for predicting bearing life [1,2]. However, estimating the parameters of Weibull distribution more accurately requires numerous sets of failure data [3]. Moreover, a large number of run-to-failure experiments is unaffordable and will take a long time to acquire data.
• This dataset contains vibration and temperature data related to the bearing life that can occur in rotating machines. Therefore, this dataset can be used to verify the performance of the newly developed life prediction or prognosis of the rotating machine based on deep learning theories [4].
• In particular, this article includes the microscopic analysis of the ball bearing through an accelerated life test. Also, by providing vibration and temperature data during all periods of the accelerated life tests, performance comparison for RUL prediction may be additionally performed according to various data locations.

## 2 Background

This dataset was established for deep learning based life prediction research. Unlike other researches, it is very difficult to obtain run-to-failure data because it is very time-consuming process for acceleration life test and it is difficult to adjusting make degradation curve through ball bearing failure. To solve this problem, we design ball bearing testbed for acceleration life test. To fatigue the bearing, a vertical load of 600 kg (5.88 kN) and an axial load of 300 kg (2.94 kN) were applied. The bearing was rotated at a constant speed of 1770–1780 RPM until bearing failure. We collected vibration, and temperature data during accelerated life test. This dataset is measured based on mechanical engineering knowledge in accordance with ISO international standards. This dataset can be used for the verification of newly-developed learning-based prognostic methods.

## 3 Data Description

This dataset consists of vibration, and temperature data. Vibration, and temperature data are collected during the acceleration life test. To make acceleration testing, an axial load of 300 kg (2.94 kN) and a vertical load of 600 kg (5.88 kN) were applied to the ball bearing. The main motor rotates at a rated rotating speed of 1770–1780 RPM.

Vibration data were measured using two accelerometers (PCB 352C34) at bearing housing in the x-direction and y-direction, simultaneously. A thermocouple (K-type) was installed on the surface of the bearing. NI9234 and NI9211 modules were used for collecting vibration, and temperature, respectively. The testbed operates at a constant speed of 1780 RPM, the rotating frequency is approximately 29.6 Hz. Additionally, the data sampling rate of vibration data using PCB 352C34 with NI9234 can calculated by their specification [5]. The data sampling rates are calculated as 51.2 kHz, 25.6 kHz, 17.067 kHz, …, 1.652 kHz based on Eq. (1) where data sampling rate (*F_s_*), internal master frequency (*F_M_*) and integer value (*n* = 1,2,3,…,31).

(1) $$F_{s}=\frac{F_{M}÷256}{n}$$

However, the PCB 352C34 covers up to 40 kHz (±10% accuracy) based on sampling theory [6]. Therefore, vibration and temperature data were collected at a sampling frequency of 25.6 kHz. This dataset was collected for 128 working hours from the normal state. After 128 working hours, the temperature and vibration level exceeded 85 °C (Celsius) and 9 m/s^2^ [7]. Then, the accelerated bearing life test was terminated.

The collected vibration and temperature data are stored in CSV files at hourly intervals. The data file contains four columns, namely ‘vibration (x_direction), ‘vibration (y_direction)’, ‘temperature (bearing)’, and ‘temperature (Atmospheric)’. The unit of the vibration data is the ‘gravitational constant (g)’ (1 *g* = 9.80665 m/s^2^). The unit of the temperature data is ‘Celsius (°C)’.

## 4 Experimental Design, Materials and Methods

**Fig. 1:**
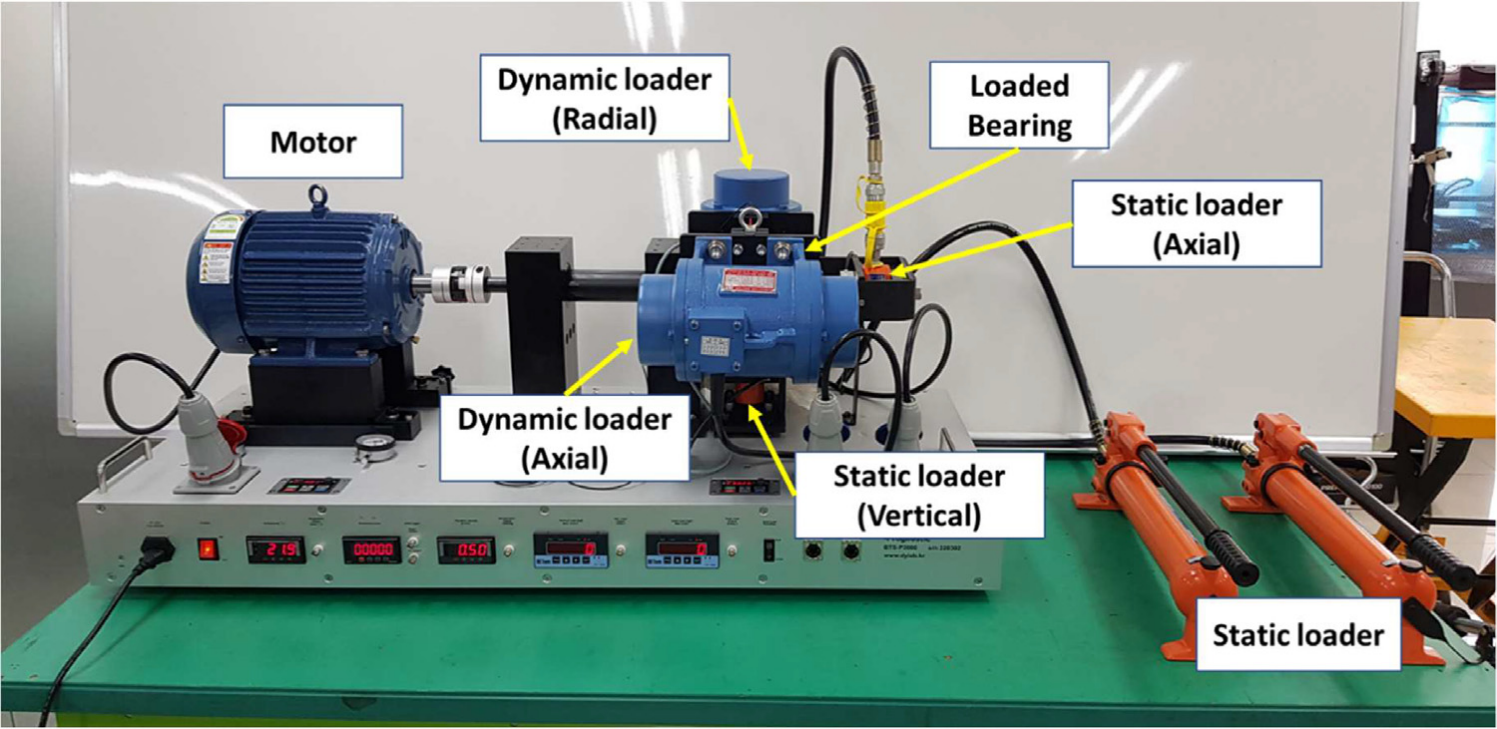
Layout of the bearing testbed for acceleration test.

The testbed consists of a three-phase induction motor, dynamic/static loader, and bearing housing as shown in Fig. 1. The three-phase induction motor rotates at a rated speed of 1770–1780 RPM. This dataset uses standardized steel NSK bearing (NSK 6205) with a ball diameter (*d*) of 7.90 mm, a pitch diameter (*D*) of 38.5 mm, contact degree angle (*θ*) of zero degrees, and the number of balls (*N*) is 9. Steel NSK bearing (NSK 6205) supports static load of 7.95 kN, and dynamic load of 14 kN. Therefore, a load was applied to the bearing using a static loader (vertical) and static loader (axial). To accelerate bearing fatigue, the axial load of 300 kg (2.94 kN) is added and the vertical load of 600 kg (5.88 kN) is added. The bearing operated for five minutes and rest for 15 min. The bearing failed at 128 working hours. To collect the dataset, a total of two accelerometers (PCB 35234) were installed in the x- and y-directions of bearing housings. Thermocouple (K-type) was installed in the bearing housing to measure the bearing temperature.

**Fig. 2:**
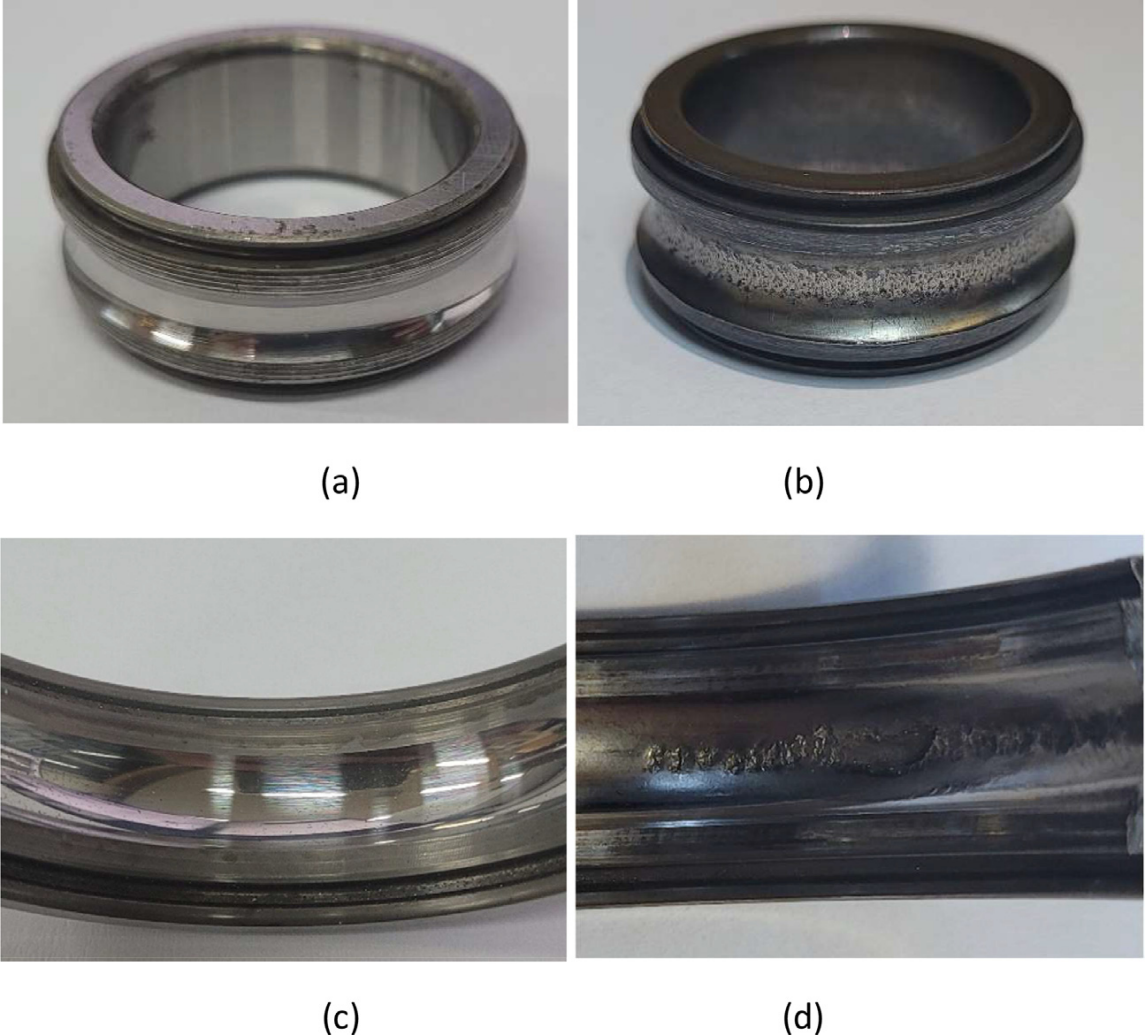
Status of Bearing: (a) inner race (normal), (b) inner race (after 128 working hour), (c) outer race (normal), and (d) outer race (after 128 working hour).

**Fig. 3:**
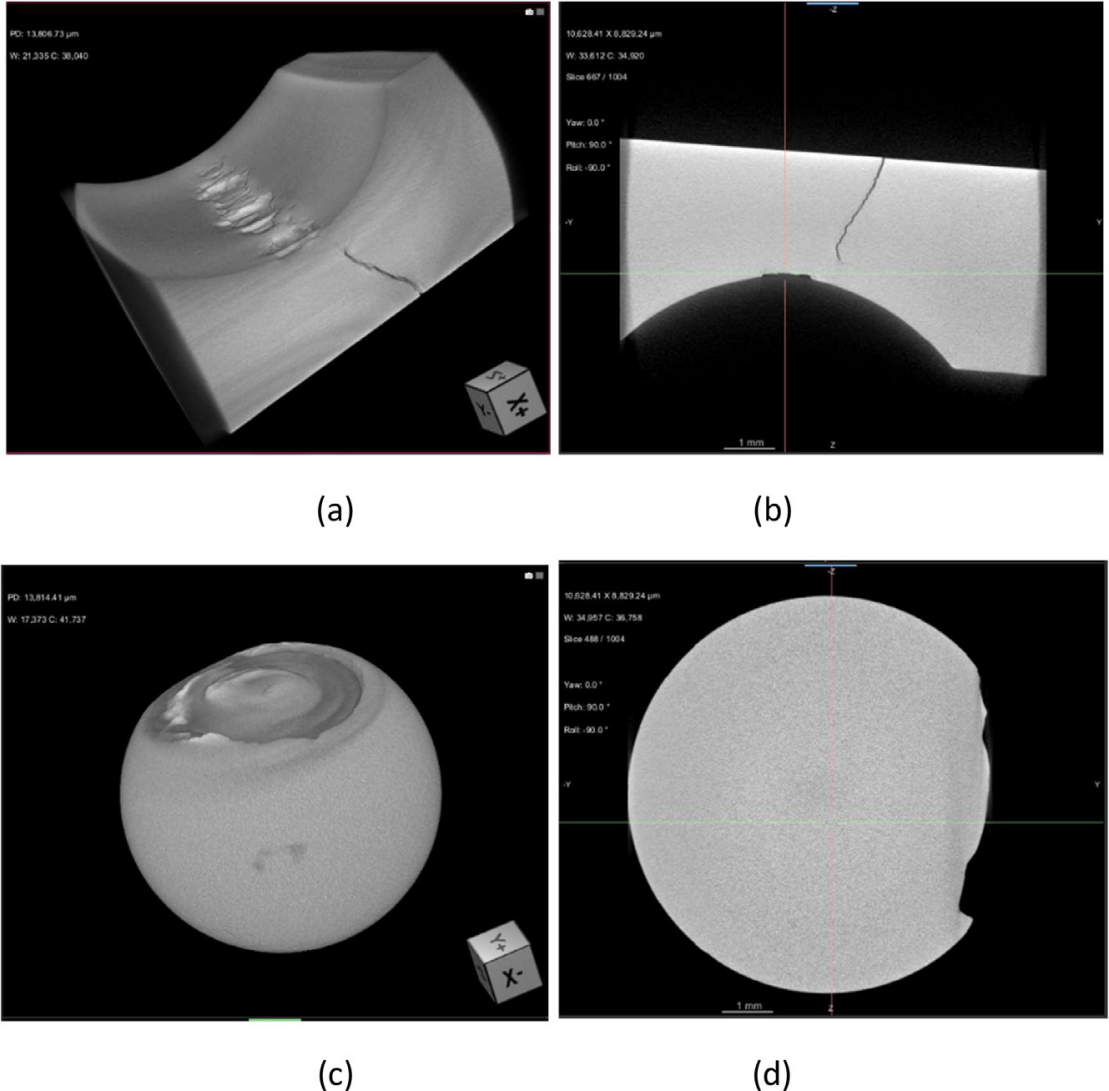
Analysis from X-ray microscope after 128 working hour: (a) outer race (full-view), (b) outer race (section-view), (c) ball (full-view), and (d) ball (section view).

**Fig. 4:**
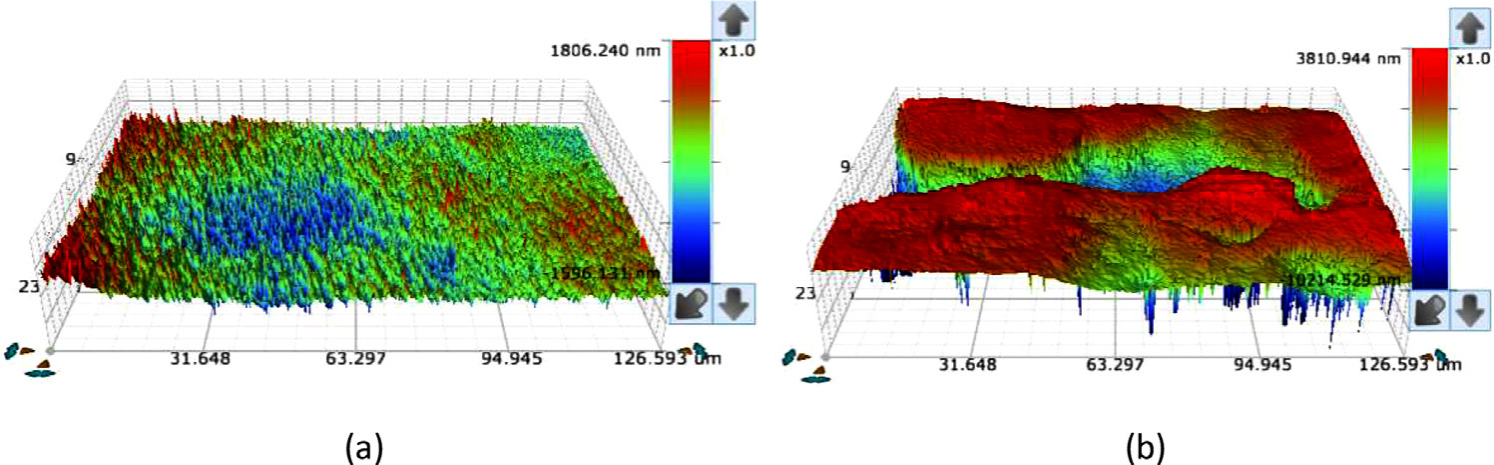
3D profiles from X-ray microscope: (a) outer race (normal), and (b) outer race (after 128 working hour).

After 128 working hours, the bearings failed as shown in Fig. 2. The inner and outer races of the bearing are normal (Fig. 2(a) and (c)). On the other hand, spalls appeared in the inner and outer races (Fig. 2(b) and (d)). As a result of X-ray analysis of the damaged bearing (Figs. 3 and 4), it was confirmed that cracks occurred in the outer race, and it was confirmed that the ball fault also occurred.

**Fig. 5:**
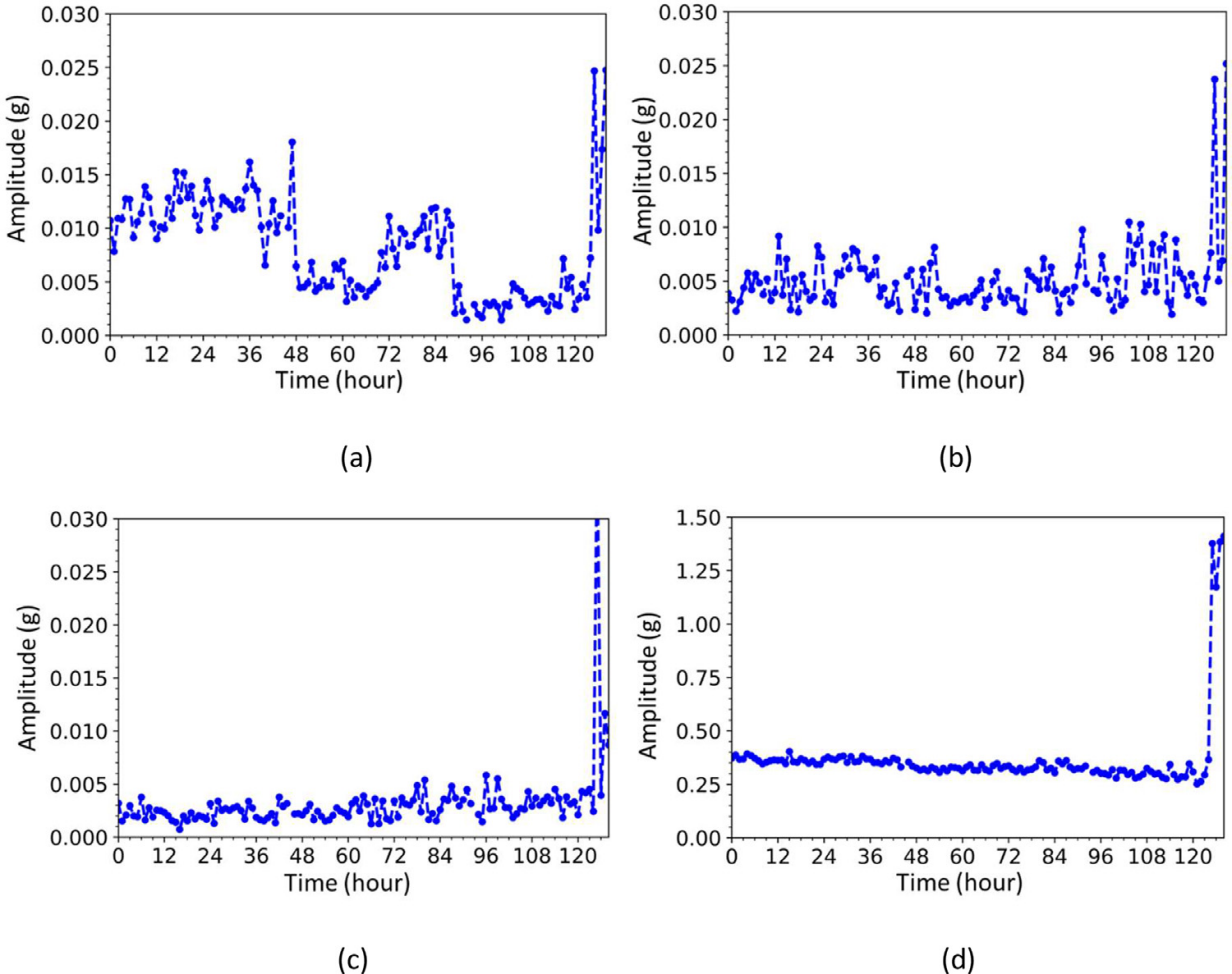
Vibration graph over time: (a) ball pass frequency of the inner race, (b) ball pass frequency of the outer race, (c) ball spin frequency, and (d) root mean square.

**Fig. 6:**
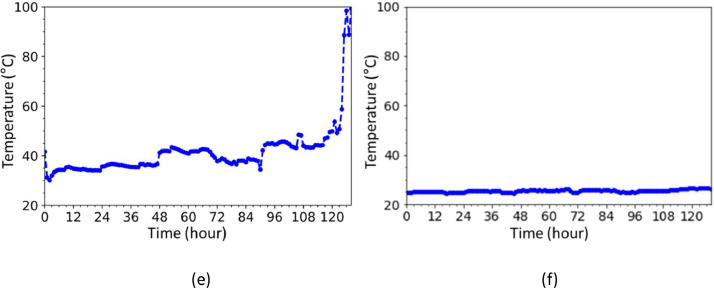
Temperature graph over time: (a) bearing, and (b) outdoor.

As shown in Fig. 5, when the vibration data and bearing temperature data according to each time file were analyzed, it was confirmed that the vibration energy value corresponding to the ball pass frequency of inner race (BPFI) component gradually increased and then decreased. It can be seen that this is reflected the physical meaning of a bearing movement. Indeed, the spall fault makes big vibration emission at first, and then the spall fault moving to a particle, and gradually lowering the vibration value. Also, the temperature level increase after bearing failure as shown in Fig. 6. In the end, as the vibration value increased again after failure, it was confirmed that the bearing was damaged, which was consistent with the results of the X-ray analysis.

## Limitations

None.

## Ethics Statement

Human Lab., Center for Noise and Vibration Control Plus, Department of Mechanical Engineering, Korea Advanced Institute of Science and Technology, Daejeon, South Korea has given the consent that the datasets may be publicly-released as part of this publication.

## CRediT authorship contribution statement

**Wonho Jung:** Conceptualization, Methodology, Software, Validation, Visualization, Writing – original draft. **Sung-Hyun Yun:** Data curation, Validation, Investigation. **Yong-Hwa Park:** Funding acquisition, Writing – review & editing, Supervision.

## Data Availability

Ball Bearing Vibration, and Temperature Run-to-Failure Dataset (Original data) (Mendeley Data)

## References

[1] Deutsch J., He D. (2017). Using deep learning-based approach to predict remaining useful life of rotating components. IEEE Trans. Syst., Man, Cybernet.: Syst..

[2] Huang R., Xi L., Li X., Liu C.R., Qiu H., Lee J. (2007). Residual life predictions for ball bearings based on self-organizing map and back propagation neural network methods. Mech. Syst. Signal. Process.

[3] Williams T., Ribadeneira X., Billington S., Kurfess T. (2001). Rolling element bearing diagnostics in run-to-failure lifetime testing. Mech. Syst. Signal Process.

[4] Ali J.B., Fnaiech N., Saidi L., Morello B.C., Fnaiech F. (2015). Application of empirical mode decomposition and artificial neural network for automatic bearing fault diagnosis based on vibration signals. Appl. Acoust..

[5] Documentation of NI-9234 specifications, National Instruments official site. https://www.ni.com/docs/ko-KR/bundle/ni-9234-specs/page/specs.html, 2024 (Accessed 24 March 2020).

[6] Documentation of PCB 352C34 specifications, PCB PIEZOTRONICS official site. https://www.pcb.com/products?m=352c34, 2024 (Accessed 24 March 2020).

[7] Documentation of ISO 10816, ISO official site. https://www.iso.org/obp/ui/#iso:std:iso:10816:-8:ed-1:v1:en, 2024 (Accessed 24 March 2020).

